# Face Adaptation—Investigating Nonconfigural Saturation Alterations

**DOI:** 10.1177/20416695211056362

**Published:** 2021-12-06

**Authors:** Ronja Mueller, Sandra Utz, Claus-Christian Carbon, Tilo Strobach

**Affiliations:** 1 Department of Psychology, 236368 Medical School Hamburg, Hamburg, Germany; 2 Bamberg Graduate School of Affective and Cognitive Sciences (BaGrACS), 14310 University of Bamberg, Bamberg, Germany; 3 Department of General Psychology and Methodology, 14310 University of Bamberg, Bamberg, Germany; 4 Research Group EPÆG (Ergonomics, Psychological Æsthetics, Gestalt), Bamberg, Germany

**Keywords:** face adaptation, face perception, face memory, non-configural face information, color information, saturation information

## Abstract

Recognizing familiar faces requires a comparison of the incoming perceptual information with mental face representations stored in memory. Mounting evidence indicates that these representations adapt quickly to recently perceived facial changes. This becomes apparent in face adaptation studies where exposure to a strongly manipulated face alters the perception of subsequent face stimuli: original, non-manipulated face images then appear to be manipulated, while images similar to the adaptor are perceived as “normal.” The face adaptation paradigm serves as a good tool for investigating the information stored in facial memory. So far, most of the face adaptation studies focused on configural (second-order relationship) face information, mainly neglecting non-configural face information (i.e., that does not affect spatial face relations), such as color, although several (non-adaptation) studies were able to demonstrate the importance of color information in face perception and identification. The present study therefore focuses on adaptation effects on saturation color information and compares the results with previous findings on brightness. The study reveals differences in the effect pattern and robustness, indicating that adaptation effects vary considerably even within the same class of non-configural face information.

## Introduction

Major theories propose that humans identify faces by matching them against mental representations stored and categorized in long-term memory (Bruce & Young, 1986). Different lighting conditions, perspectives, and fluctuant variations of faces often cause them to appear differently than their mental representations stored in memory. However, despite these ongoing changes, we perceive our environment as stable, and we can reliably identify faces, especially if they are personally familiar (Carbon, 2008). This performance is ensured by continuously adjusting our mental representations to the dynamics of the environment. This adjusting process, which is commonly referred to as adaptation, can lead to a strong misperception in subsequently perceived faces. By integrating face alterations into our face representations, we perceive unaltered faces as being manipulated (since they do not match anymore with our updated representations; e.g., Carbon et al., 2007). Specifically, these items are perceived as being manipulated in the opposite direction to the adaptor. Face images slightly manipulated in the direction of the adaptor, however, would be perceived as “normal” or “natural” since the images correspond with the updated representation (e.g., Webster & MacLin, 1999).

Unlike the related phenomenon of afterimages (e.g., the color inverted image that continues to appear after the exposure to a visual stimulus) that is supposed to rely on retinal sensitivity adjustments, adaptation effects most probably address also later stages of the visual pathway (e.g., Carbon & Ditye, 2011; Webster & MacLeod, 2011). These high-level contributions are indicated by a significant number of face adaptation studies, revealing effects that are highly robust over time (e.g., Carbon et al., 2007; Carbon & Ditye, 2011, 2012; Strobach et al., 2011) and also transferable from one person's face to another person's face (e.g., Carbon et al., 2007). Simple retinal sensitivity adjustments (and also recency effects; see, e.g., Glanzer & Cunitz, 1966; Mueller et al., 2021; Tan & Ward, 2000) cannot explain the robustness and transferability of face adaptation effects but rather suggest a processing on a representational memory basis (e.g., Carbon et al., 2007; Carbon & Ditye, 2011).

Although many different types of face information have already been investigated using the adaptation paradigm (e.g., face information regarding expression, age, ethnicity, or gender; Fox & Barton, 2007; Lai et al., 2012; Little et al., 2008; Webster et al., 2004), most of the face adaptation studies focused on configural face information so far (i.e., second-order spatial relations between facial features; Maurer et al., 2002; Piepers & Robbins, 2012; for a review, see Strobach & Carbon, 2013). Studies investigating adaptation effects on nonconfigural face information (i.e., information that does not affect any relational aspects of the face), such as color, are rather rare. This is remarkable as there is mounting evidence revealing the importance of this type of information in face perception and identification. A loss of color, for example, might lead to recognition impairments, while different color alterations reveal information about the ethnic background or the health and emotional status of a person (e.g., Lee & Perrett, 1997; Levin & Banaji, 2006; Re et al., 2011; Thorstenson et al., 2019). Different color dimensions are, therefore, interpreted and perceived very differently. Due to the lack of adaptation studies on color, it is not yet clear, whether these differences in perception are also reflected in face memory. The current study aims to fill the gap of lacking non-configural color adaptation studies by focusing on face adaptation effects on saturation alterations and comparing these effects with effects of alternative color information (i.e., brightness).

### Face Adaptation Effects on Color

One of the few existing face adaptation studies on color information is the study by Little et al. (2012). The authors used so-called color or shape transformed antifaces as adaptors. Antifaces are defined as faces that possess traits opposite to a specific identity in terms of a facial average (i.e., by defining the differences between a specific face and an average face that is created of many different faces, an antiface can be computed that lies on the mirror opposite). Antifaces transformed in color, for example, would be opposite in color composition compared to the original face image (e.g., originally blond hair would be probably darker and dark skin would become brighter in an antiface). The authors were able to show that participants' ability to recognize the corresponding celebrity in the average face was improved when adaptors displaying color or shape transformed antifaces of the celebrities are presented in advance. The revealed effects were stronger for shape than for color-transformed antiface adaptors. This improved recognizability is probably based on a shift of mental representations. When integrating the face traits of the antifaces into mental representations, the representations are altered toward these traits and thus also the representation of the average face. When looking at the image of the (original and unaltered) average face, it would then be perceived as having face traits opposite to the antiface since the original average face image does not correspond to the updated representation of the average face anymore. This way, participants would tend to identify the corresponding celebrity in the average face image (for further theoretical input on face representation see the literature on the so-called “face space”; e.g., Lewis, 2004; Valentine, 1991, 2001; Valentine et al., 2016). The study shows that color information is most likely integrated into mental representations. However, this study does not allow conclusions about specific color dimensions and their adaptation effects.

An adaptation study by Mueller et al. (2021) focused on one specific color dimension when investigating face adaptation effects. We used celebrity faces strongly manipulated in brightness (either strongly decreased or increased) as adaptors and presented slightly manipulated face images (also decreased or increased in brightness) and non-manipulated images in the test phase. When asking participants to choose the original (i.e., non-manipulated) image from the presented test images, participants showed a clear bias in their selection by choosing the test image that was more similar to the adaptor seen before (i.e., participants showed an adaptation effect). Since experiments, using nonface or inverted face stimuli altered in brightness as adaptors, were not able to evoke adaptation effects, the observed adaptation effects on celebrity faces seem to be face-specific. Furthermore, by varying the time interval between adaptation and test phase we investigated the robustness of effects. Since they lasted 300 ms, 3 s, and up to 5 min (maybe even up to 50 min across the entire experiment), the effects appear to be not just based on simple retinal sensitivity adjustments but on short-term or maybe even long-term memory processes. Thus, we assume that the observed adaptation effects also affect the face representations stored in memory. We found further evidence that the adaptation effects on brightness alterations affect rather late stages of the visual pathway and facial memory by applying different transfer dimensions in our adaptation paradigm. Different transfer dimensions are implemented by not only (1) presenting the identical face image in the adaptation and test phase (*pictorial* level), but also (2) presenting either different images of the same identity (*structural* level) or (3) even different identities in the adaptation and test phase (*cross-identity* level; for a review on other studies applying these transfer dimensions see Strobach & Carbon, 2013). Although the adaptation effects on the transfer levels structural and cross-identity were attenuated compared to the pictorial level, we observed an adaptation effect on all three transfer levels to a certain degree. This transferability of effects indicates that representations not just of a specific identity are altered through adaptation but also superordinate concepts that subsume different identities with common underlying structures (for more information on rather abstract face representations, see e.g., Carbon & Ditye, 2011; Leopold et al., 2001; Mueller et al., 2020; Strobach & Carbon, 2013).

To the knowledge of the authors, brightness information was the only type of specific color information investigated in face adaptation studies so far. However, previous findings suggest that different color dimensions are perceived and interpreted very differently. Tan and Stephen (2013), for example, were able to show that facial redness, compared to brightness, must be somehow perceived as more salient. Moreover, different color dimensions seem to be associated with different characteristics. Increased saturation, for example, is most likely associated with a person's emotional or health state (Re et al., 2011; Thorstenson et al., 2019). Brightness information, however, seems to refer to the person's relative skin tone when deducting contextual conditions (Levin & Banaji, 2006; Mueller et al., 2021). The few existing face adaptation studies (i.e., the studies by Little et al., 2012; Mueller et al., 2021) seem to be not yet able to capture this differentiation in the perception of facial color, and thus it is not clear yet whether differences in the perception of specific color dimensions are also reflected in mental face representation. It is, therefore, worth taking a more differentiated look at color information. Consequently, the present study aims to provide a greater variability of color adaptation studies by examining color information in the form of saturation alterations. Next to hue and brightness, saturation is one of the three core dimensions traditionally characterizing a perceived color (e.g., Burns & Shepp, 1988; Indow & Kanazawa, 1960). As the studies by Re et al. (2011) and Thorstenson et al. (2019) revealed (see above), saturation seems to provide essential information about a face. An investigation of the retention of saturation information in face memory and the comparison with brightness could therefore be of interest.

### The Aim of the Study

The present study investigates (1) whether adaptation to saturation alterations (increased and decreased saturation) generally occurs, (2) whether adaptation effects are robust over time, (3) whether saturation adaptation effects are face-specific, and (4) whether adaptation effects on saturation alterations differ in their magnitude from effects on brightness alterations. Five experiments were conducted in total. Four of these experiments were based on the study procedure of Mueller et al. (2021), but investigated adaptation effects on saturation alterations. Three of these four experiments used celebrity images as adaptor stimuli. The other experiment was performed using non-face adaptation images (scrambled faces) that were manipulated in saturation. This experiment was conducted to clarify whether possible adaptation effects on saturation are selective for faces or whether they are general color aftereffects that also occur when presenting nonface stimuli. The three experiments using celebrity adaptation images altered in saturation differed in their time interval between adaptation and test phase. By increasing the time interval between the adaptation and test phase from very short to a relatively long time interval, the durability of possible adaptation effects can be investigated. The study outcomes could provide information about the processing level of adaptation effects on saturation information. They could clarify whether possible adaptation effects on saturation are processed on a sensory level only or if they affect also higher levels of the visual pathway and thus potentially also face representations (long-term adaptation affects probably higher levels of the visual pathway and thus more likely also face representations; see, e.g., Carbon et al., 2007; Carbon & Ditye, 2011). In all of these experiments, we implemented three different transfer levels: pictorial, structural, and cross-identity (for more detailed information see the description above of the study by Mueller et al., 2021). The implementation of these transfer dimensions might provide insights into the representational level at which adaptation effects can occur. To compare possible adaptation effects on saturation with adaptation effects on brightness, one further study was conducted using adaptation and test stimulus material manipulated in saturation and brightness. Hereby, the strength of the effects of both color alterations could be directly evaluated. In this experiment, only the pictorial dimension was tested.

The adaptation stimulus manipulation was performed differently depending on the group the participants belonged to: they were exposed to stimuli either (1) strongly decreased in saturation/brightness, (2) without any manipulation, or (3) increased in saturation/brightness. In all experiments, a two-alternative-forced-choice (2AFC) test was presented subsequently, instructing the participants to select the veridical image (i.e., the unaltered image) out of two different image versions, displaying a non-manipulated image and an image version slightly manipulated in saturation/brightness (i.e., decreased or increased saturation/brightness). In case an adaptation would occur, we expect the strong manipulations seen in the adaptation phase to be integrated into the face representations stored in the participants’ memory leading to an alteration of the face representations toward the adaptor. Non-manipulated images would then be perceived as being manipulated in the opposite direction to the adaptor (e.g., after adapting to an image increased in saturation, a non-manipulated image would appear decreased in saturation, and vice versa). Hence, it would be expected that the participants select the face version that is more similar to the adaptor when being exposed to the 2AFC test.

## Experiment 1

Experiment 1 aims to find out whether adaptation effects occur for nonconfigural saturation alterations. Hence, celebrity images strongly manipulated in saturation were presented as adaptors (i.e., decreased, original, and increased saturation) and we tested adaptation effects on the pictorial, structural, and cross-identity levels. Between the adaptation and test phase, we implemented a short time interval of 300 ms.

### Method

#### Participants

Forty-eight undergraduate students from the Medical School Hamburg (30 females, 18 males, *M*_age_: 24.2 years, range 20–38 years) participated in Experiment 1.

An a priori power analysis that was based on a 3 (between participants) by 3 (within participants) factor mixed-design analysis of variance (ANOVA) revealed a minimal sample size of *N*  =  45 (Faul et al., 2007). Given an *α*  =  0.05 and a test power of (1 − *β*)  =  0.90 the design was able to detect a medium–large effect size *f* of 0.25 (Cohen, 1988). Previous studies in our lab context mostly revealed medium–large up to medium-to-large adaptation effects when using similar, familiar (celebrity) faces (e.g., Carbon & Ditye, 2011; Mueller et al., 2021). Typically, we have not found small or very small effects, even if we used longer-termed experimental designs. This was the reason for assuming medium–large effects. To provide a balanced study design, we increased the sample size to *N*  =  48, resulting in a test power of 0.93.

Participants were either rewarded with money (12 Euros) or recruited as part of teaching course requirements. All participants were naïve regarding the purpose of the experiment. Participants' vision was assessed with the Freiburg Visual Acuity and Contrast Test (Bach, 1996). Furthermore, a short version of the Ishihara color test (Ishihara, 1917) was employed to test for anomalies in color perception. Participation in the subsequent testing was limited to participants with normal or corrected-to-normal vision, according to these tests. We randomly assigned participants to one of three participant groups (presenting either adaptation stimuli decreased in saturation, nonmanipulated images, or images increased in saturation). All participants provided informed written consent. The study was approved by the ethics board of the Medical School Hamburg (date of application: March 9, 2017) and conducted according to the guidelines of the Declaration of Helsinki.

#### Apparatus and Stimuli

**Figure 1.**
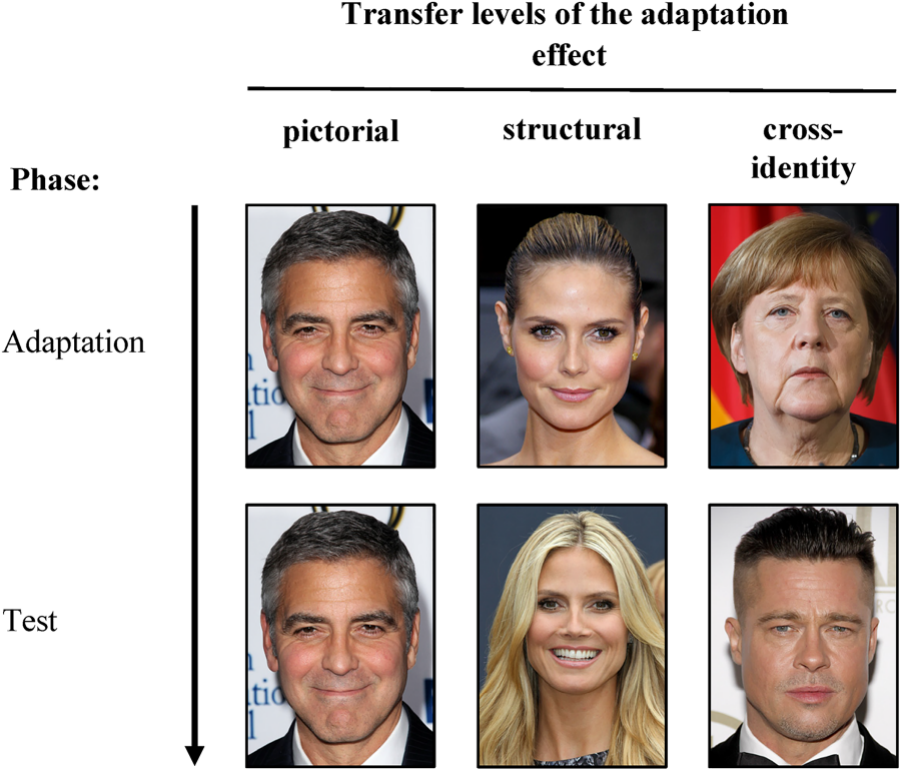
Illustration of the transfer levels pictorial, structural, and cross-identity. Each column represents a different transfer level. The pictorial transfer level describes a test condition where the images of the adaptation and test phase are pictorially identical (e.g., George Clooney as the adaptor and test stimulus). The structural transfer level is characterized by presenting different images of the same identity (e.g., different images of Heidi Klum as the adaptor and test stimulus) and on the transfer level cross-identity, images of the adaptation and test phase even displayed different identities (Angela Merkel as the adaptor and Brad Pitt displayed as the test stimulus). The images were not used in the original study but are displayed here for illustrative purposes only. The figure is taken from Mueller et al. (2021). Permissions and image licenses have been obtained from the copyright holders (Sources: ©Drop of Light/Shutterstock.com, Tinseltown/Shutterstock.com, s_bukley/Shutterstock.com).

The apparatus and procedure closely follow Mueller et al. (2021). Seventy celebrity names were randomly collected from newspaper articles and by asking colleagues. For each celebrity, a photograph was selected and presented in a subsequent survey (92 participants; 66 females, mean age: 21.7 years, range: 18–30 years) in which participants were instructed to judge the celebrity's familiarity on a 5-point Likert scale (1  =  *unfamiliar*; 5  =  *very familiar*) and write down the celebrities’ names (if known). The 30 celebrities^1^ whose familiarity was rated highest and named most often were included as stimulus material in the subsequent tests. Two photos (A and B; other than the photos in the survey) were selected for each of the 30 celebrities, fulfilling the following criteria: the celebrity's full face was presented in frontal view, with a straight gaze, no glasses, and no hair covering any facial features (e.g., eyes, nose, or mouth). Furthermore, the images were of high resolution. All 30 celebrities were randomly assigned to 1 of 3 celebrity groups. Since two photos (A and B) were selected for each celebrity, there were a total of six different stimulus sets. These sets were used to create the three different transfer levels, pictorial, structural, and cross-identity, that differed in terms of the overlapping information between images presented in the adaptation and test phase (all transfer levels of the within-participants factor *transfer* are illustrated in Figure 1).

**Figure 2.**
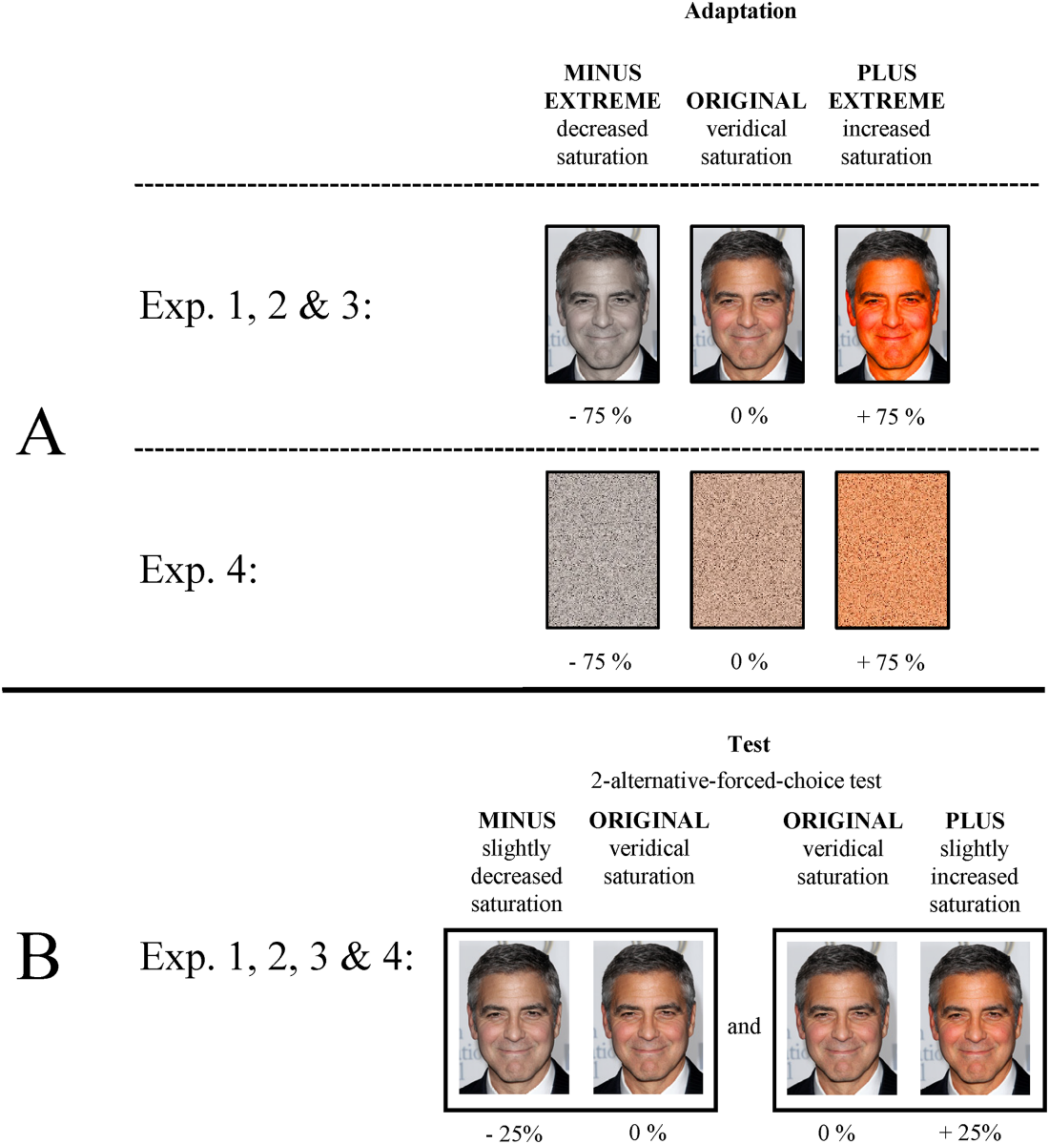
The different image versions are illustrated by the face of George Clooney. (a) Illustration of the different adaptor versions in Experiments 1 to 4. Experiments 1 to 3 use the same adaptation images. Experiment 4 applies scrambled versions of the adaptor images used in Experiments 1 to 3. (b) Illustration of the image versions during the test phase in Experiments 1 to 4. Within the 2-alternative-forced-choice (2AFC) test, two images are displayed (the original image together with an image either decreased or increased in saturation). The position of the images (centered left or centered right) alternated. The illustration is adapted from Mueller et al. (2021). Permissions and image licenses have been obtained from the copyright holders (Sources: ©Drop of Light/Shutterstock.com).

All images were manipulated by altering the saturation of the presented faces (without hair) in Adobe Photoshop CC (Version 19.0). Different levels of manipulations were applied resulting in five different image versions (manipulation of the saturation by −75%, −25%, 0%, + 25%, and +75%), as illustrated in Figure 2. The size of the images was ∼330  ×  412 pixels. Depending on which group the participants belonged to (between-participants factor), they either saw an unaltered image (0%, *ORIGINAL*), an image strongly decreased in saturation (−75%, *MINUS EXTREME*) or an image with a strongly increased saturation ( + 75%, *PLUS EXTREME*) as an adaptor. The adaptation stimuli of the MINUS EXTREME and PLUS EXTREME participant groups could obviously be identified as manipulations. During the test phase, the participants were exposed to two image versions, with one image always displaying the ORIGINAL photo and the other image showing a slightly manipulated version with either −25% saturation (MINUS) or +25% (PLUS). The term ORIGINAL image should be treated with caution since it might not display a completely unaltered image. Previous manipulations (although not obvious) carried out by the photographer, for example, may have been applied, and thus the identity might be presented in an edited way. Therefore, the term ORIGINAL image rather indicates that the image has not been altered in saturation. Moreover, since possible manipulations are not obvious, the face images should sufficiently reflect our accumulated experience with the presented identities. The experiment was created in Experiment Builder 2.2.1 (SR Research) and run on a Lenovo PC with a 23″ computer screen running at a resolution of 1,920  ×  1,080 pixels.

#### Procedure

**Figure 3.**
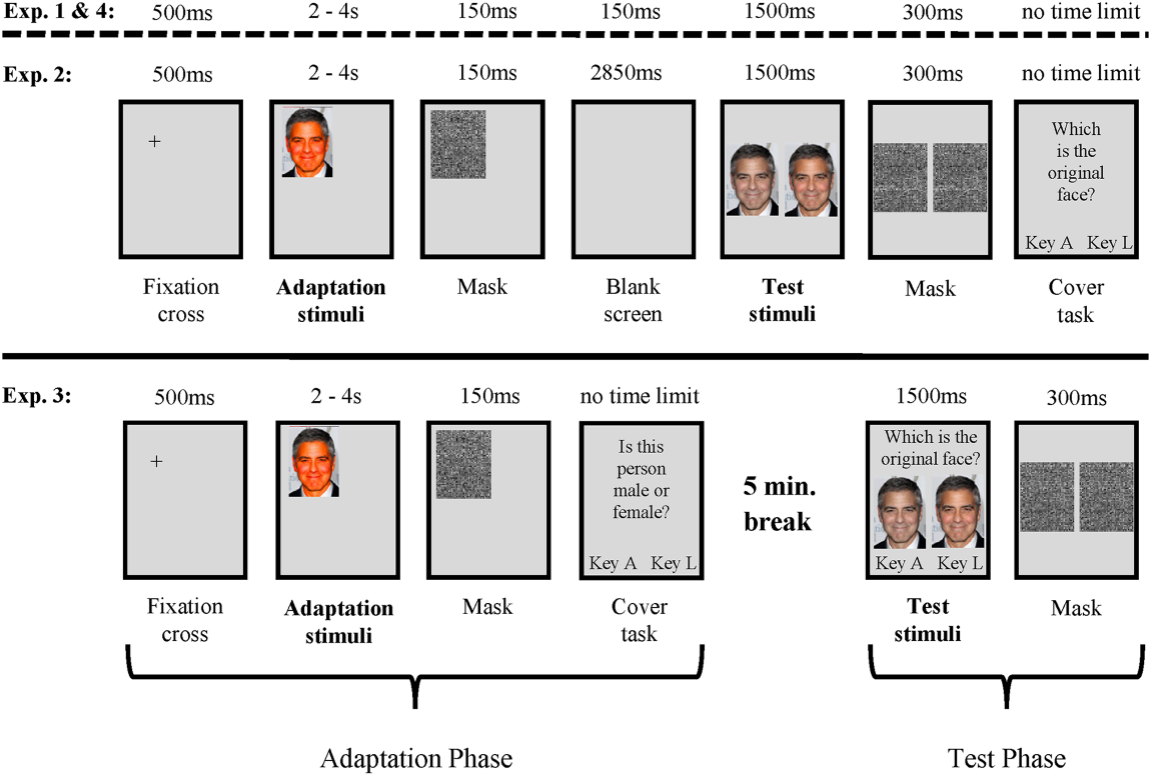
Schematic illustration of the trial structure of Experiments 1 to 4. Trials of Experiments 1 and 4 are identical regarding their timing. Experiments 1 and 4 differ only in their stimulus material (see Figure 5). In Experiment 2, the length of the interstimulus interval was increased from 300 ms (Experiments 1 and 4) to 3,000 ms (Experiment 2), by increasing the presentation duration of the blank screen from 150 ms (Experiments 1 and 4) to 2,850 ms (Experiment 2). In Experiment 3, the adaptation and test phases are presented separately (with a 5 min break in between). An image of George Clooney is used here as an example. Adapted from Mueller et al. (2021); permissions and image licenses have been obtained from the copyright holders (Sources: © Drop of Light/Shutterstock.com).

As illustrated in Figure 3, each trial included both the adaptation and test phase. All trials started with a fixation cross, presented in the center of the subsequent stimulus position for 500 ms. Subsequently, the adaptor was displayed, showing either the ORIGINAL image or one of the two extreme manipulations (MINUS EXTREME or PLUS EXTREME), depending on which group the participants belonged to (see Figure 2 for an illustration). Following the procedure of Carbon et al. (2007), the adaptor images were presented in one of six different screen positions (top-left, top-center, top-right, bottom-left, bottom-center, or bottom-right), to control for retinal adaptation effects. Moreover, by displaying the adaptor images for either 2, 3, or 4 s, we increased the variability of the task with the aim of reducing fatigue effects without decreasing the inspection time of the adaptation stimuli. Each adaptor image was presented four times at each presentation time and twice on each screen position. Thus, each individual adaptor was shown 12 times during the whole experiment. During adaptation, participants encountered only two (out of six) stimulus sets of celebrity faces (e.g., image set A or B of two [out of three] different celebrity groups). The third group of celebrity faces was not displayed during adaptation. For each participant, the displayed stimulus sets were determined in advance, whereby the presentation order of the stimuli was randomized within the experiment. The presented stimulus sets were balanced across participants of one adaptation group. Subsequently to the adaptor, participants were exposed to a backward mask for 150 ms, to eliminate possible afterimages (Turvey, 1973). Following the mask, a blank screen appeared for 150 ms, creating (together with the presented mask) an interstimulus interval of 300 ms between adaptation and test stimuli (i.e., the 2AFC test).

Once the blank screen was presented, the 2AFC was asked for, displaying two image versions of the same photo (that only differed in their saturation) for 1,500 ms (always the ORIGINAL with either a MINUS image version [−25% saturation] or a PLUS image version [+25% saturation]). The position of both presented images (centered left or centered right) was randomized and balanced throughout the experiment. While only two celebrity groups were presented during adaptation, all three groups were shown during the test phase, to implement all three transfer levels. Depending on the transfer level, the test images were either identical to the preceding adaptor (pictorial, e.g., trials presenting images of image set A of celebrity group 1 as adaptors and test stimuli), represented different images of the same identity (structural, e.g., trials where image set A of celebrity group 2 was displayed during adaptation and image set B of the same celebrity group as test stimuli) or displayed identities from the celebrity group not shown during adaptation (cross-identity, e.g., trials where image set A of celebrity group 1 was displayed during adaptation and image set A of celebrity group 3 as test stimuli). The adaptation phase of the cross-identity transfer level was formed out of the two adaptor sets that were used for the pictorial and structural transfer levels (e.g., image sets 1A and 2A). Half of each adaptor set (i.e., half of the pictorial image set [e.g., half of the image set 1A] and half of the structural image set [e.g., half of the image set 2A]) was displayed in the adaptation phase of the cross-identity transfer level (i.e., half of the pictorial and structural image sets [e.g., half of set 1A and 2A] served as adaptor stimuli and the set not shown during adaptation [e.g., image set 3A] was presented in the cross-identity test phase). Across all transfer levels, the saturation of the adaptors was kept constant within each participant group. As for the adaptation phase, the image sets presented in the test phase were determined in advance and balanced across participants. The trial order was randomized within the experiment.

Following the two test images, a backward mask appeared for 300 ms to eliminate any afterimages and thus a prolonged exposure to the test stimuli. Subsequently, participants were required to select the test image they considered to be the veridical one (instructions were saying in German: “Which is the original face?”). To mark their selection, participants pressed a specific button on a keyboard that corresponded to the image position (key “A” for images presented on the left side, key “L” for images presented on the right side). Following the procedure of Carbon et al. (2007), the participants were informed to base their decision on their knowledge about the celebrity (e.g., images that one has encountered in the media) and not on the images inspected in the experiment. This way, participants were encouraged to access the mental representation stored in memory, when finding the “veridical” image in the test phase. Moreover, since the previously displayed adaptation stimuli were obviously strongly manipulated, it should have been clear to the participants that they were not supposed to base their decision on these adaptor images when being asked to select the nonmanipulated image in the test phase. The test phase had a total of 360 trials. After presenting half of the trials (i.e., 180 trials) a break occurred, offering the participants time to relax and informing them that they had completed half of the test. The participants independently started the second half of the test.

After the adaptation and test phases, the participants were again presented with the previously seen celebrity images. This time, they were instructed to judge the celebrities according to their familiarity (instructions were: “Are you familiar with this celebrity from the media?”). Participants responded with “yes” or “no” by pressing again the buttons “A” or “L” on the keyboard, respectively. The ratings were used to exclude trials presenting celebrities that were unknown to the participants since alterations of face representations through adaptation are expected to be more pronounced for faces that are familiar (as familiar faces are already represented in memory; for a comparison of adaptation effects on familiar vs. unfamiliar faces, see Hills & Lewis, 2012). Altogether, the experiment lasted about 60 min.

### Results and Discussion

On average, 98.9% of the face stimuli were rated as familiar (individually ranging from 86.7% to 100%) and thus included in further analysis. Moreover, all individual outliers (i.e., response times slower than 3SD above the individual mean response time) of each participant as well as trials with a response time faster than 200 ms, were excluded. The dependent variable of interest was the average test face selection in the 2AFC. The test face selection is an indicator of whether a prior observation of, for example, strongly manipulated images, causes a shift in face perception. The variable was scored according to the degree of manipulation of the selected test images: A score of −25 was assigned to the MINUS images, a score of 0 to the ORIGINAL images, and a score of +25 to the PLUS images.^2^

**Figure 4.**
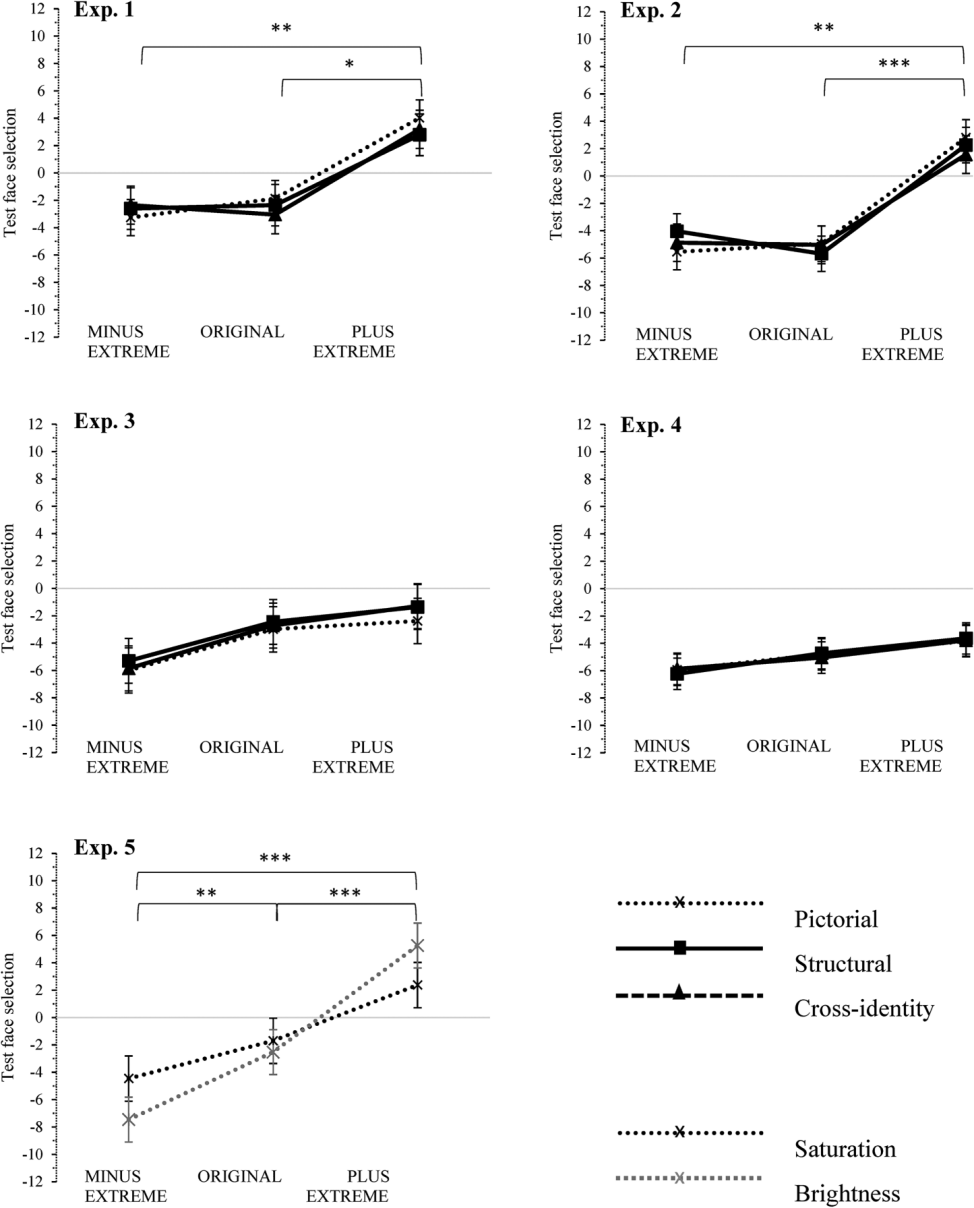
Illustration of the interaction between adaptation group and transfer level for Experiments 1 to 5 and of the interaction between the adaptation group and color type for Experiment 5. The *y*-axis reflects the degree of saturation (and brightness in Experiment 5) of the selected test images in the 2-alternative-forced-choice (2AFC; in %). Error bars represent  ±  1 standard error of the mean.

According to the experimental design, a two-way, mixed-design ANOVA was calculated with the between-participants factor adaptation group (MINUS EXTREME, ORIGINAL, and PLUS EXTREME) and the within-participants factor transfer level (pictorial, structural, and cross-identity). There was a main effect for the adaptation group [*F*(2, 45)  =  6.39, *p*  =  .004, $\eta_{p}^{2}$ =  .221] with the means: *M*_MINUS EXTREME_  =  −2.74 (*SD*  =  5.02), *M*_ORIGINAL_  =  −2.42 (*SD*  =  3.06) and *M*_PLUS EXTREME_  =  3.34 (*SD*  =  7.26). Bonferroni-corrected comparisons revealed significant differences between MINUS EXTREME and PLUS EXTREME: *p*  =  .008, *d*  =  −0.97 and between ORIGINAL and PLUS EXTREME: *p*  =  .013, *d*  =  −1.05. We could not reveal a significant difference between MINUS EXTREME and ORIGINAL*: p*  =  1.000, *d*  =  −0.06. Since the adaptation group PLUS EXTREME reveals a significant bias in the participant's selection toward an increased saturation compared to the other two adaptation groups, an adaptation effect on saturation is indicated at least for the *PLUS EXTREME* adaptation group. The main effect of transfer level was not significant (*F*[2, 90] < 1, *p*  =  .612, $\eta_{p}^{2}$ =  .011), neither was there an interaction between transfer level and adaptation group (*F*[4, 90]  =  1.59, *p*  =  .184, $\eta_{p}^{2}$ =  .066, see Figure 4 (Experiment 1) for an illustration). Thus, it seems that the transfer level has no impact on the obtained adaptation effects.

## Experiment 2

Since the results of Experiment 1 indicate face adaptation effects on saturation alterations (at least for the increased saturation condition), we wanted to clarify the processing level of these adaptation effects by varying the temporal factors of the following two experiments (Experiments 2 and 3). While very short time intervals between the adaptation and test phase only allow predictions about more or less sensory-based (retinotopic) effects, adaptation effects using relatively longer time intervals indicate processing on higher cognitive levels. In Experiment 2, the time interval (interstimulus interval) between the adaptation and test phase was consequently extended from 300 ms (in Experiment 1) to 3,000 ms. All other characteristics remained the same from the previous experiment.

### Method

#### Participants

Forty-eight undergraduate students from the Medical School Hamburg participated in Experiment 2. The sample size was the same as in Experiment 1. The requirements and conditions were identical to those in Experiment 1. No participant of this experiment had taken part in Experiment 1.

#### Apparatus and Stimuli

The apparatus and stimuli were identical to those used in Experiment 1.

#### Procedure

Experiments 1 and 2 were identical in their procedure except that the length of the interstimulus interval was increased from 300 ms (Experiments 1) to 3,000 ms. This was done by increasing the presentation duration of the blank screen from 150 ms (Experiment 1) to 2,850 ms. Thus, after the presentation of the adaptor, a backward mask was displayed for 150 ms, and subsequently, a blank screen appeared for 2,850 ms, leading to an interstimulus interval of 3,000 ms.

### Results and Discussion

On average, 97.0% of the face stimuli were rated as familiar (individually ranging from 66.7% to 100%) and thus included in further analysis. The general outlier analyses, as well as the 2AFC analysis, were similar to Experiment 1.

A two-way, mixed-design ANOVA was calculated with the between-participants factor adaptation group (MINUS EXTREME, ORIGINAL, and PLUS EXTREME) and the within-participants factor transfer level (pictorial, structural, and cross-identity). There was a main effect for the adaptation group (*F*[2, 45]  =  11.19, *p* < .001, $\eta_{p}^{2}$ =  .332) with the means: *M*_MINUS EXTREME_  =  −4.82 (*SD*  =  4.06), *M*_ORIGINAL_  =  −5.22 (*SD*  =  3.62) and *M*_PLUS EXTREME_  =  2.21 (*SD*  =  6.69). Bonferroni-corrected comparisons revealed significant differences between MINUS EXTREME and PLUS EXTREME: *p*  =  .001, *d*  =  −1.27 and between ORIGINAL and PLUS EXTREME: *p* < .001, *d*  =  −1.39. We could not reveal a significant difference between MINUS EXTREME and ORIGINAL*: p*  =  1.000, *d*  =  0.11. Since the adaptation group PLUS EXTREME reveals a significant bias in the participant's selection toward an increased saturation compared to the other two adaptation groups, an adaptation effect on saturation is indicated at least for the PLUS EXTREME adaptation group. The main effect of transfer level was not significant (*F*[2, 90] < 1, *p*  =  .79, $\eta_{p}^{2}$ =  . 005), neither was there an interaction between transfer level and adaptation group (*F*[4, 90]  =  1.75, *p*  =  .147, $\eta_{p}^{2}$ =  .072 see Figure 4 [Experiment 2]). Thus, it seems that the transfer level has no impact on the adaptation effects.

## Experiment 3

Since the results of Experiment 2 indicate that face adaptation effects on saturation alterations (at least for the increased saturation condition) last several seconds, the following experiment aims to determine whether the effects are even more robust, lasting not only seconds but several minutes. We did so by changing the trial-wise procedure (applied in the previous two experiments) into a block-wise procedure, where the adaptation and test phase are presented in two different blocks, separated by a 5 min break (adopted from Carbon et al., 2007). An occurrence of adaptation effects after five minutes would indicate involvement of higher cognitive processes than just sensory processing.

### Method

#### Participants

Forty-eight undergraduate students from the Medical School Hamburg (36 females, 12 males, *M*_age_: 22.9 years, range 19–30 years) participated in Experiment 3 and were tested individually. The sample size was the same as in Experiments 1 and 2. The requirements and conditions were identical to those in Experiments 1 and 2. No participant of this experiment had taken part in Experiment 1 or 2.

#### Apparatus and Stimuli

The apparatus and stimuli were identical to those used in Experiment 1.

#### Procedure

Experiment 3 differed from the first two experiments in that the adaptation and test phases were administered in different blocks, separated by a 5 min break. As Figure 3 illustrates, a trial within the adaptation phase started by presenting a fixation cross for 500 ms, followed by the adaptation stimulus. Depending on the group the participants belonged to, they were either exposed to a MINUS EXTREME image, an ORIGINAL image, or a PLUS EXTREME image. As in the two experiments before, the adaptation stimuli were displayed on six different screen positions for either 2, 3, or 4 s. Screen positions and timing conditions were balanced across trials. Subsequently to the adaptor stimulus, a backward mask appeared (150 ms) before a cover task was presented (saying in German: “Is this person a male or female?”). A selection was made by pressing a button on a keyboard (key “A”  =  male; key “L”  =  female). The image sets that were used as the adaptation stimuli were determined in advance and balanced across participants. Following the adaptation phase, a 5 min break was given, in which the participant was instructed to read a geographical text. This was intended to prevent a mental recall of the faces seen before.

Subsequently, to the 5 min break, the 2AFC test was presented, displaying either an ORIGINAL and a MINUS image or an ORIGINAL and a PLUS image for 1,500 ms in each trial. As in the experiments before, the stimulus position was randomized and balanced across trials. Image sets used in the test phase represented all three celebrity groups to apply all three transfer levels (pictorial, structural, and cross-identity). The stimulus sets were balanced across participants. Following the stimulus presentation, backward masks were displayed for 300 ms. Subsequently, the participants were asked to choose the veridical image out of the two images presented in the previously seen 2AFC test. The response was indicated by pressing either button A (corresponding to the left image) or button L (corresponding to the right image) on the keyboard. The adaptation phase included 360 trials, while the test phase included 120 trials. As in the two experiments before, a familiarity rating task was conducted following the adaptation and test phase. Altogether, the experiment lasted about 60 min.

### Results and Discussion

On average, 98.7% of the face stimuli were rated as familiar (individually ranging from 70% to 100%) and thus included in further analysis. The general outlier criteria, as well as the 2AFC analysis, were similar to the previous experiments.

Based on the between-participants factor adaptation group (MINUS EXTREME, ORIGINAL, and PLUS EXTREME) and the within-participants factor transfer level (pictorial, structural, and cross-identity), a two-way, mixed-design ANOVA was calculated. We did not reveal a main effect for the factor adaptation group (*F*[2, 45]  =  1.74, *p*  =  .187, $\eta_{p}^{2}$ =  .072) nor for the factor transfer level (*F*[2, 90]  =  1.51, *p*  =  .226, $\eta_{p}^{2}$ =  .033). Also for the combination of transfer level and adaptation group (*F*[4, 90] < 1, *p*  =  .915, $\eta_{p}^{2}$ =  .011), no significant interaction could be revealed (see Figure 4 [Experiment 3] for an illustration). Thus, we could not detect an adaptation effect in Experiment 3.

## Experiment 4

Although Experiment 3 indicated that adaptation effects on saturations are not detectable after several minutes, we wanted to clarify whether the observed effects in Experiments 1 and 2 are selective for faces or whether they are general color aftereffects, that also occur when presenting nonface stimuli. Thus, in the following experiment, we apply nonface stimuli that were altered in saturation in the adaptation phase. By scrambling the adaptation faces (i.e., the entire images) presented in the previous experiments beyond recognition, the nonface stimulus material was created (Figure 2a). Hence, each adaptation face stimulus presented in the experiments before was divided into tiny little pieces and randomly assembled so that a homogeneous color area was formed (a so-called scrambled face) that reflected the average color of the particular face stimulus. In the test phase, however, we used the same stimuli as in the experiments before (i.e., celebrity faces). In case the observed adaptation effects in Experiment 1 and 2 are face-specific, adaptation effects in the following experiment should be reduced. Since the images displayed in the adaptation phase no longer represent any faces, an application of the transfer levels pictorial and structural is no longer possible. For the sake of consistency between all reported experiments, however, all three transfer levels (i.e., pictorial, structural, and cross-identity) shall be deployed.

### Method

#### Participants

Forty-eight undergraduate students from the University of Bamberg (39 females, 9 males, *M*_age_: 23.3 years, range 18–36 years) participated in Experiment 4 and were tested individually. All other requirements and conditions were identical to those in Experiments 1 to 3.

#### Apparatus, Stimuli, and Procedure

The apparatus and procedure of Experiment 4 were identical to Experiment 1. The experiments only differed in their adaptation stimuli. The adaptation stimuli displayed in Experiment 4 were scrambled versions of the images used in Experiment 1 (Figure 2a). The stimuli were scrambled to such an extent that the faces were no longer recognizable.

### Results and Discussion

On average, 96.3% of the face stimuli were rated as familiar (individually ranging from 76.7% to 100%) and thus included in further analysis. The general outlier analyses, as well as the 2AFC analysis, were similar to the previous experiments.

Based on the between-participants factor adaptation group (MINUS EXTREME, ORIGINAL, and PLUS EXTREME) and the within-participants factor transfer level (pictorial, structural, and cross-identity), a two-way, mixed-design ANOVA was calculated. There was neither a main effect for the factor adaptation group (*F*[2, 45]  =  1.11, *p*  =  .338, $\eta_{p}^{2}$ =  .047) nor for the factor transfer level (*F*[2, 90] < 1, *p*  =  .992, $\eta_{p}^{2}$ < .001). Also for the combination of transfer level and adaptation group (*F*[4, 90] < 1, *p*  =  .952, $\eta_{p}^{2}$ =  .006), no significant interaction could be revealed (see Figure 4 [Experiment 4] for an illustration). Thus, we did not obtain any adaptation effect regarding saturation alterations of scrambled faces in Experiment 4 based on our experimental design.

To determine whether adaptation effects are significantly larger after short delays than after longer delays, as well as in faces than in scrambled faces, we further calculated a two-way ANOVA with the between-participants factor experiment (Experiments 1–4) and the within-participants factor transfer level (pictorial, structural, and cross-identity). Since adaptation effects were only apparent in the PLUS EXTREME adaptation group (i.e., the increased saturation condition), only the PLUS EXTREME adaptation groups of all experiments were compared. There was a significant main effect for the factor experiment (*F*[3, 60]  =  3.96, *p*  =  .012, $\eta_{p}^{2}$ =  .165). Bonferroni-corrected comparisons revealed significant differences between Experiments 1 (300 ms interstimulus interval) and 4 (using scrambled faces): *p*  =  .023, *d*  =  1.141. However, although it approached conventional levels of significance, there was neither difference between Experiment 2 (3 s interstimulus interval) and the scrambled face Experiment 4 (*p*  =  .084) nor between Experiment 3 (5 min interstimulus interval) and the scrambled face Experiment 4 (*p*  =  1.000). Furthermore, there was neither a main effect for the factor transfer level (*F*[2, 120] < 1, *p*  =  .822, $\eta_{p}^{2}$ =  .003) nor for the interaction between transfer level and experiment (*F*[6, 120]  =  1.40, *p*  =  .220, $\eta_{p}^{2}$ =  .065). It should be noted that from an experimental perspective, only the comparison between Experiment 1 and the scrambled face Experiment 4 is reasonable, since these two experiments are the only ones with the same experimental set-up (trial-wise procedure with a 300 ms interstimulus interval). The comparisons of the other experiments should be therefore interpreted with caution.

## Experiment 5

Experiment 3 indicated that adaptation effects on saturation alterations are not observable after several minutes. Thus, adaptation effects on saturation seem to be not as robust as adaptation effects on brightness, which still occurred after 5 min (see Mueller et al., 2021). To see if there are also differences in the degree of adaptation effects, the following experiment directly compared adaptation effects on saturation with adaptation effects on brightness alterations. Thus, stimuli altered in saturation and stimuli altered in brightness were presented within one paradigm.

### Method

#### Participants

Forty-eight undergraduate students from the Medical School Hamburg (34 females, 14 males, *M*_age_: 23.4 years, range 19–31 years) participated in Experiment 5. All other requirements and conditions were identical to those in Experiments 1 and 2.

#### Apparatus and Stimuli

**Figure 5.**
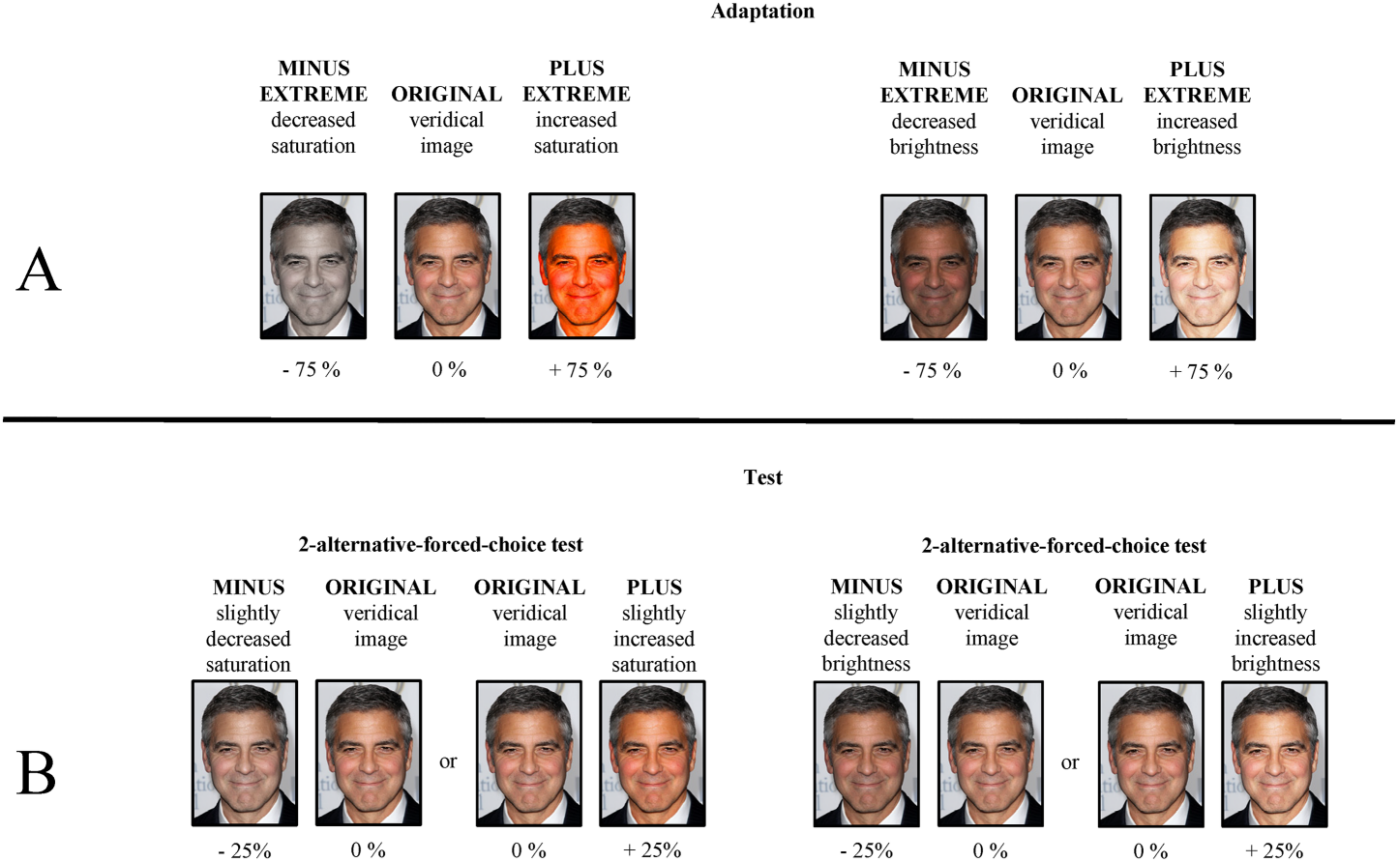
The different image versions in Experiment 5 are illustrated by the face of George Clooney. (a) Illustration of the different adaptor versions. (b) Illustration of the image versions during the test phase. Within the 2-alternative-forced-choice (2AFC) test, two images are displayed (the original image together with either an image with decreased saturation/brightness or an image with increased saturation/brightness). Adapted from Mueller et al. (2021). Permissions and image licenses have been obtained from the copyright holders (Sources: ©Drop of Light/Shutterstock.com).

The stimulus material of Experiment 5 consisted of the same celebrity images as in Experiment 1 but altered not only in saturation but also in brightness (within-participants factor). Different levels of manipulations were applied, resulting in five different image versions per manipulation type (manipulation of the saturation and brightness by −75%, −25%, 0%, +25%, and +75%), as illustrated in Figure 5. As in Experiment 1, the size of the images was ∼330  ×  412 pixels. Depending on which group the participants belonged to (between-participants factor), they either saw an unaltered image (ORIGINAL), an image with a strongly decreased color manipulation (−75% of either saturation or brightness, MINUS EXTREME), or an image with a strongly increased color manipulation (+75% of either saturation or brightness, PLUS EXTREME) as an adaptor. During the test phase, the participants were exposed to two image versions, with one image always displaying the ORIGINAL photo and the other image showing a slightly manipulated version with either a −25% manipulation (MINUS) of saturation or brightness or a +25% manipulation (PLUS) of saturation or brightness. The color manipulation (saturation or brightness) presented in the test phase corresponded to the color manipulation the participants inspected in the previous adaptation phase. The experiment was created in Experiment Builder 2.2.1 (SR Research) and run on a Lenovo PC with a 23″ computer screen running at a resolution of 1,920  ×  1,080 pixels.

#### Procedure

The timing conditions of Experiment 5 were identical to Experiment 1. Adaptation and test phases were again applied within single trials (see Figure 3). Within a trial, stimuli of only one color type (saturation or brightness) were presented (in the adaptation as well as in the test phase). Trials displaying saturation alterations and trials displaying brightness alterations were presented in two different blocks, whereby the presentation order of the stimuli within one block was randomized within the experiment. Each block consisted of 180 trials, so that the whole experiment consisted of 360 trials. Each participant was exposed to only one image set. Thus, only the pictorial transfer level was applied, so that the test images were identical to the adaptation image seen before (except the different color alterations applied to the images). All celebrity groups and all image sets were applied and balanced across participants of one adaptation group. All other conditions were the same as in Experiment 1. Altogether, the experiment lasted about 60 min.

### Results and Discussion

On average, 97.4% of the face stimuli were rated as familiar (individually ranging from 76.7% to 100%) and thus included in further analysis. The general outlier analyses and the 2AFC analysis were identical to the previous experiments.

Based on the between-participants factor adaptation group (MINUS EXTREME, ORIGINAL and PLUS EXTREME) and the within-participants factor color type (with factor levels: saturation and brightness), a two-way, mixed-design ANOVA was calculated. There was a main effect for the adaptation group (*F*[2, 45]  =  29.19, *p* < .001, $\eta_{p}^{2}$ =  .565) with the means: *M*_MINUS EXTREME_  =  −5.96 (*SD*  =  2.72), *M*_ORIGINAL_  = −2.11 (*SD*  =  2.90), and *M*_PLUS EXTREME_  =  3.82 (*SD*  =  5.12). Bonferroni-corrected comparisons revealed significant differences between MINUS EXTREME and PLUS EXTREME: *p* < .001, *d*  =  −2.43 and between ORIGINAL and PLUS EXTREME: *p* < .001, *d*  =  −1.37. There was also a significant difference between MINUS EXTREME and ORIGINAL: *p*  =  0.014, *d*  =  −1.52. The main effect of color type was not significant (*F*[1, 90] < 1, *p*  =  .751, $\eta_{p}^{2}$ =  .002), neither was there an interaction between color type and adaptation group (although results approach conventional levels of significance; *F*[4, 90]  =  3.085, *p*  =  .056, $\eta_{p}^{2}$ =  .121, see Figure 4 [Experiment 5]). Thus, adaptation effects seem to be demonstrated across both alteration conditions (i.e., decreased and increased saturation/brightness alterations).

This finding would make the results on saturation clearly different from those in Experiments 1 and 2. In the first two experiments, adaptation effects could only be demonstrated for the increased saturation condition compared to the other conditions. It should be noted that the interaction approaches conventional levels of significance. When analyzing the interaction more closely (irrespective of the probably lacking interaction) and focusing on saturation only, multiple comparisons reveal a significant difference between the ORIGINAL and PLUS EXTREME groups (*p*  =  .043, *d*  =  −0.67), but not between the ORIGINAL and MINUS EXTREME groups (*p*  =  .166, *d*  =  −0.65). This indicates that there would be an adaptation effect only for the increased saturation condition but not for the decreased condition, when analyzing the results of saturation and brightness separately. However, when analyzing multiple comparisons even further, adaptation effects did not differ between both color types (MINUS EXTREME adaptation group *p*  =  .084; ORIGINAL adaptation group *p*  =  .628; PLUS EXTREME adaptation group *p*  =  .095). Thus, there seem to be no differences in the adaptation on saturation and brightness.

The fact that the interaction effect and also the direct comparisons of saturation and brightness did not reach conventional levels of significance (although some of the effects are not far from being significant) could be related to the different design of the experiment (e.g., only the pictorial transfer condition was applied). The slightly different design of the study could possibly lead to lower test power. A post hoc power analysis of the interaction of adaptation group and color type indeed revealed a rather low test power of (1 − *β*)  =  0.73 (3  ×  2 factor design, given an effect size *f* of 0.37, an *α* of 0.05, and *N* of 48). Thus, the missing interaction of adaptation group and color type might be caused by a low test power. Potentially, the adaptation pattern of the first experiments (i.e., adaptation effect for the increased but not the decreased saturation condition) would also emerge in Experiment 5 if the test power had been higher.

## General Discussion

Face adaptation seems to be a useful tool to investigate how faces are perceived and stored in memory. Up to now, mainly configural face information has been subject to adaptation studies often neglecting nonconfigural face information, such as color. To the knowledge of the authors, brightness was the only type of specific color information investigated in face adaptation studies so far. The reported experiments, therefore, aim to provide a greater variability in the literature of adaptation studies on color by examining other color information in the form of saturation alterations.

### Existence of Adaptation Effects on Saturation Alterations

The results clearly show that adaptation effects exist for at least increased saturation alterations (i.e., the PLUS EXTREME adaptation group shows a significant difference to the ORIGINAL and MINUS EXTREME condition; Experiments 1 and 2). Unlike original or decreased face versions, stimuli strongly increased in saturation lead to a perceptual bias when inspecting subsequent faces. This is reflected in a more frequent selection of images with a slightly increased saturation in the 2AFC for participants belonging to the PLUS EXTREME adaptation group (the average selection of test faces in this group is *M*  =  3.34 for Experiment 1 and *M*  =  2.21 for Experiment 2). Thus, after being exposed to strongly increased saturation images, participants tend to perceive a slightly increased saturation image as being the nonmanipulated (original) image. The significant main effect of the factor adaptation group of Experiment 5 (saturation and brightness are both included) surprisingly revealed adaptation effects for both alteration conditions (i.e., increased and decreased)—although this result has to be questioned due to the almost significant interaction of color type and adaptation group and the low test power of this experiment. Also, when analyzing the interaction and focusing on saturation only, the same pattern as in the first two experiments (i.e., adaptation effect for the increased but not the decreased saturation condition) can be observed. Hence, unlike effects on brightness adaptation, effects on saturation seem to occur only in one direction (i.e., for increased saturation alterations).

However, this result should also be evaluated regarding the other two adaptation groups (i.e., MINUS EXTREME and ORIGINAL). Graphically, a shift of the ORIGINAL adaptation group in the negative direction can be observed in all studies. One-sample *t*-tests revealed that the shift was also statistically significant in all experiments (one-tailed, *ps* < .035). Thus, participants seeing nonmanipulated faces or even nonmanipulated scrambled images as the adaptor (ORIGINAL) somehow tend to choose images decreased in saturation (and also brightness in Experiment 5) as the veridical image in the test phase. The causes for this bias are not clear yet. It might be that participants that did not adapt to any extreme image version (i.e., MINUS EXTREME or PLUS EXTREME images) somehow perceive an increase in saturation (or brightness) as more evident as a decrease. This way, participants of the ORIGINAL adaptation group would more easily identify slightly increased test images (PLUS) as being manipulated and would therefore reject them when choosing the veridical face. The slightly decreased version (MINUS) and the original image (ORIGINAL), however, are maybe not as easy to distinguish, so that participants would more often select decreased images in test trials where a decreased image and the original image are presented. This response tendency (i.e., rejecting all increased images while selecting decreased images at times) could result in an overall bias of the ORIGINAL adaptation group into the negative range. Moreover, assuming that this perceptual bias is becoming also apparent in the adaptation phase, the difference between ORIGINAL and MINUS EXTREME adaptor images would probably be perceived as being smaller compared to the ORIGINAL and PLUS EXTREME images as well. This, however, could explain the missing adaptation effects in the decreased adaptation group. Future studies should, therefore, investigate possible anomalies in the perception of saturation (and also brightness). Furthermore, studies should adjust the saturation (or brightness) level of the adaptors, so that differences between adaptation groups would be perceptually equal. This way it could be revealed whether the missing adaptation effects on decreased saturation is caused by experimental factors or because adaptation on decreased saturation is generally not possible (for a discussion on the perception of different brightness levels, see Mueller et al., 2021).

Since adaptation effects could not be provoked using nonface stimuli (unrecognizable scrambled face stimuli manipulated in saturation), Experiment 4 indicates that the adaptation effects on saturation are maybe face-specific. Moreover, the direct comparison of Experiment 1 (300 ms interstimulus interval) and Experiment 4 (scrambled faces) also revealed a significant difference in adaptation, supporting the assumption that adaptation to saturation is qualitatively different in faces versus nonface stimuli (the nonsignificant difference for Experiment 2 compared to the scrambled face experiment might be due to the different experimental design). Hence, face adaptation effects on saturation might not just reflect general color aftereffects. Instead, they seem to be contextual dependent, meaning that they are linked to the specific context of faces. Accordingly, face adaptation effects on saturation might occur only when using adaptors displaying faces. It should be noted, however, that also the large variation in adaptation and test images could have led to this result. Moreover, the scrambled faces representing homogeneous color areas might attract less attention than human faces (for comments on how faces specifically attract attention, see, e.g., Palermo & Rhodes, 2007). Less attention on the adaptor stimuli could possibly have resulted in a failure to adapt (previous studies, however, suggest that attention may not be a decisive factor for adaptation; see Stein et al., 2012, revealing adaptation effects when using interocular suppression). It could be possible though that images that are more similar in their composition to the applied face images (e.g., in contrast and complexity) would evoke adaptation effects when altered in saturation. Therefore, future studies should apply other nonface stimuli than scrambled faces (e.g., objects or inverted faces; see, e.g., Mueller et al., 2021).

Experiment 5 did not reveal any differences between adaptation effects on saturation compared to brightness. This result, however, should be questioned, since the interaction almost reached conventional levels of significance, and the test power of the experiment should be considered as rather low. Hence, it might be that there is a difference in the adaptation of saturation and brightness. Brightness would in this case cause stronger adaptation effects than saturation (PLUS EXTREME adaptation group: *M*_Saturation_  =  2.38, *M*_Brightness_  =  5.27). Accordingly, the observed differences in the perception of different color dimensions (see, e.g., Re et al., 2011; Tan & Stephen, 2013; Thorstenson et al., 2019; or the “Face Adaptation Effects on Color” section) are maybe even reflected in the retention of these colors in face memory. To validate this assumption further adaptation studies on saturation and brightness and other color dimensions should be conducted.

### Processing Level of Adaptation Effects on Saturation

Experiment 2 revealed that the reported adaptation effects in Experiment 1 still occur after a delay of 3,000 ms. However, adaptation effects did not appear when applying a delay of 5 min. Thus, the adaptation effects on saturation alterations do not seem to be very robust. Unlike adaptation effects on brightness alterations, which last up to 5 min (Mueller et al., 2021), effects on saturation seem to be more transient. However, the adaptation effects still seem to be retained for up to 3,000 ms, which might indicate an involvement of the short-term memory. Nevertheless, since the effects do not seem to affect the long-term memory, it is not clear whether adaptation operates on a representational memory or a rather sensory basis. Future studies should investigate how long the adaptation effects on saturation can last precisely (anything between 3,000 ms and 5 min) and whether the effects decrease over time.

Previous adaptation studies revealed a continuous decay of adaptation effects, suggesting some kind of “resetting” mechanism of the mental representation (see, e.g., Carbon et al., 2007; Carbon & Ditye, 2011; Carbon & Leder, 2005; Mueller et al., 2021; Strobach et al., 2011). It could be possible that the resetting of saturation information somehow occurs faster than for other face information. It could be, for example, that saturation information is integrated into the representation but because of an automatic resetting process (e.g., due to the robustness of the original representation) that occurs very quickly, adaptation effects on saturation are relatively transient. A quick resetting of saturation could possibly be related to the lower importance of this kind of information for face identification. The possibly lower importance for face identification could be due to the variant nature of saturation information. Compared to configural face information, which is associated with invariant personal characteristics (such as identity, sex, or ethnicity), saturation information would be rather labeled as a variant type of face information, since it is often associated with a person's emotional or health state (e.g., Re et al., 2011; Thorstenson et al., 2019). Because of its rather variant nature, saturation information might not be stored in long-term memory, since it does not provide very stable information for identifying a face. The presumably higher robustness of brightness adaptation effects compared to saturation (Mueller et al., 2021, found adaptation effects on brightness up to 5 min) might be related to a rather invariant nature of brightness information (facial brightness probably refers to a person's skin tone and thus maybe even to a person's ethnicity). Hence, the robustness of facial color information (and possibly face information in general) might be related to the information's variability.

The different robustness of saturation and brightness could also be interpreted in accordance with a very popular model of face recognition and retention. Bruce and Young (1986) described a functional model which outlines the processes occurring when recognizing and processing a face. The model distinguishes between a *pictorial code,* a *structural code,* and a so-called *face recognition unit* (the model describes further structures for identity processing but since they are not relevant here they shall not be explained further). The pictorial code describes picture-dependent processing and representation of a face (comparable with the pictorial transfer level) while the structural code reflects a more abstract face processing and representation that is based on the specific structure of a face. It enables face recognition despite changes in the depiction of the face (e.g., despite a different head angle, expression) and is thus comparable with a processing on the structural transfer level applied in our study. However, there is one decisive difference between the structural transfer level applied in our study and the structural code proposed by Bruce and Young (1986): adaptation effects occurring on a structural transfer level often serve as an indicator that a representation of a specific identity is accessed (see section “Face Adaptation Effects on Color”). Face processing in terms of the structural code proposed by Bruce and Young (1986), however, does not refer to a specific identity. The facial structure is processed and analyzed with respect to, for example, the age or gender of the person, but an attribution to a specific identity is not made before subsequent processing stages were pursued. In the very next stage, which is labeled the “face recognition unit,” the assignment of the specific face structure to a specific identity is performed, although only in terms of a feeling of knowing of the target person. In terms of this model, the different robustness of saturation and brightness could be due to processing at different processing levels. Since saturation information might not be very relevant for face identification (due to its variant nature), it could be possible that saturation information is only processed in terms of the structural code without affecting a specific identity. Since brightness information might be more relevant for face identification due to its more invariant nature, it might be processed even in terms of the face recognition unit and thus in regard to a representation of a specific identity.

Further research is needed, however, to substantiate these assumptions. Thus, upcoming studies should investigate other variant and invariant face information to see if the observed differences between invariant and variant face information also become apparent in other types of information than saturation and brightness. Moreover, paradigms should be developed to investigate the identity–specificity of adaptation effects on a structural level. This could be used to examine whether different types of information are processed differently in the sense of Bruce and Young’s (1986) model. Furthermore, upcoming studies should investigate whether this adaptation pattern is similar for facial saturation alterations affecting other color dimensions than redness (e.g., saturation alterations that tend more toward the green or the blue). Since increased saturation leading to a greater redness is often associated with a person's emotional or health state (e.g., blushing; see Re et al., 2011; Thorstenson et al., 2019; or the section “Face Adaptation Effects on Color”), it would be interesting, if saturation alterations also occur for other (maybe nonnatural) color dimensions. The missing adaptation effects on decreased saturation might be an indication that adaptation effects rather occur for “natural” saturation alterations (a decrease in saturation leads to a loss of color which can be considered as nonnatural).

As shown by the results of Experiments 1 and 2, saturation seems to be indeed processed on a structural level (for effects of brightness on different transfer levels, see Mueller et al., 2021). The adaptation effects seem to transfer well across different images of the same identity, suggesting that the effects are not only image-specific but must affect a more abstract face representation that captures the structure of the face. Therefore, it is likely that adaptation alters the represented face structure (independent of its attribution to a specific identity). This way, adaptation effects would also occur when presenting images of the identity that differ from the adaptor. Furthermore, Experiments 1 and 2 showed that adaptation effects also transfer between different identities (cross-identity level). These results suggest that adaptation operates on a hierarchically higher level, affecting not only a specific face representation but probably superordinate category representations, such as a prototype representation or a generic face norm. Hence, by altering a specific face representation through adaptation, superordinate prototype representations would be altered too, leading to an adaptation effect when presenting other identities than the identity presented as the adaptor.

Taking into account the possible face-specificity of the observed adaptation mechanism, effects on increased saturation might be better explained by modifications of face representations than by merely perceptual or retinal processes. Simple recency effects (i.e., tendency to recall the last perceived information the best) are probably not able to account for the reported results, since the stimuli presented in the cross-identity transfer level (and to some extend also in the structural transfer level) differ tremendously (a recency effect could only be considered if the stimuli were identical). Thus, it seems likely that increased saturation information is stored in representations (whether of specific identities or as a more general face norm). Hence, adapting to strongly increased saturation alterations would cause the manipulations to be integrated into a facial representation or mental norm. By integrating the increased saturation alterations into mental representations, the initial face norm would be shifted in the direction of the adaptor (e.g., after adapting to a face increased in saturation, the face representation would be “updated” and thus shifted slightly toward the increased saturation adaptor). Following this adaptation process, images only slightly increased in saturation would then be perceived as “normal” (since the image matches the updated representation), while non-manipulated images would be perceived as being manipulated reversely (i.e., as being decreased in saturation).

### Conclusions

The reported experiments clearly revealed adaptation effects for non-configural saturation information, although they can be observed only for increased saturation alterations. Thus, adaptation effects can indeed be demonstrated for non-configural face information other than brightness. The results indicate that the adaptation to increased saturation information might be face-specific. Moreover, it is probably stored in the facial representation but maybe not in the long term. Adaptation effects on saturation, therefore, differ in their robustness (and maybe even in their strength) from adaptation effects on brightness. This difference could be based on the somewhat variant nature of increased saturation information compared to brightness information, which can be considered rather invariant. While brightness might provide information about a person's ethnicity and thus identity, increased saturation probably makes essential contributions in identifying emotional and health states. This way adaptation processes on increased saturation might even facilitate the interpretation of complex social situations by enabling a more efficient differentiation of different emotional and health states in other people. However, due to the great variability of emotional and health status, the retention of new information only for a short amount of time is very reasonable since emotional and health status may have changed already within the next moment. A flexible adaptation of the representation and a resetting to the initial representational norm, therefore, seems to be necessary. The here presented experiments indicate that face representations contain a variety of face information that seems to be stored at least partly independent from each other. Moreover, different types of face information seem to be represented with different valence, depending on the relevance the information type has for face recognition. Research on adaptation effects on different face information can help to find out more about the complex mental representation of faces. Possibly, through face adaptation research, we may one day be able to better understand the seemingly contradictory nature of mental face representations: on the one hand, they seem to be stable, ensuring fast and valid face recognition, on the other hand, they are very flexible and adapt quickly to facial changes.

## Notes

1. Musicians (Adele, Amy Winehouse, Ed Sheeran, Justin Bieber, Justin Timberlake, Katy Perry, Pink, Rihanna, and Selena Gomez), actors (Cameron Diaz, Daniel Radcliffe, Elyas M’Barek, Emma Watson, George Clooney, Jennifer Aniston, Leonardo DiCaprio, Til Schweiger, and Will Smith), politicians/head of states (Angela Merkel, Barack Obama, Donald Trump, Queen Elizabeth, Vladimir Putin), athletes (Boris Becker, Cristiano Ronaldo, and Joachim Löw), models (Heidi Klum and Paris Hilton), and entertainers (Thomas Gottschalk and Dieter Bohlen).

2. The largest possible adaptation effect toward a decreased saturation would be attained, when a participant would always select a *MINUS* image (−25), in trials presenting *MINUS* and *ORIGINAL* images in the 2AFC test and an *ORIGINAL* image (0), in trials presenting *ORIGINAL* and *PLUS* images in the 2AFC test. The largest adaptation effect toward an increased saturation would be attained by always choosing a *PLUS* image (+25) to be the veridical one in trials, displaying *PLUS* and *ORIGINAL* images and an *ORIGINAL* image (0), in trials presenting *ORIGINAL* and *MINUS* images. Since the versions of the 2AFC test trials were balanced across the experiment, the dependent variable (test face selection) can vary from −12.5 to +12.5 for each participant.
